# Remarks upon the Medicinal Plants of Cherokee, Georgia

**Published:** 1857-01

**Authors:** Robert Battey

**Affiliations:** Rome, Georgia


*Remarks upon the Medicinal Plants of Cherokee, Georgia.* By ROBERT BATTEY, of Rome, Georgia.

While this section of Georgia was occupied by the Cherokee tribe of Indians, the collection and exportation of medicinal plants and roots, together with slugs of silver, (obtained from a source now unknown) skins and venison hams were their only means of securing the requisite supplies of salt, whisky, gunpowder, calico, &c., consumed by them. One George Lavender, a white man, (who early attached himself to the Cherokees, and afterwards married, I believe, the daughter of John Ridge, one of their chiefs,) was the principal trader of the tribe. Establishing himself at the point now known as Rome, he carried on a considerable trade in the articles named, and is said to have had engaged in his service numbers of wagons, transporting these commodities to Augusta, a distance of two hundred and fifty miles, and returning with goods for his store. In this way he accumulated in some twenty years quite a large fortune. He sent to market chiefly pink root, serpentaria, senega and ginsing. I can obtain no definite data as to the annual amount thus sent off, or the relative quantities of each. Spigelia and serpentaria doubtless predominated largely. I am informed that he was in the habit of shipping spigelia with the top attached, for which he exchanged salt, powder and dry goods, allowing the Indian two cents the pound. During one season, having the monopoly of salt, he is said to have exchanged an entire sack in small lots, for slugs of native silver, weight for weight. Many marvellous tales are told of him and his traffic.

For some years prior to the removal of the Cherokees west, the supply of these plants greatly diminished, until the trade in them almost entirely ceased, and the Indians devoted themselves more to the culture of grain, which became so abundant as to be almost worthless as an article of sale. During the space which elapsed since their departure, the stock of medicinal plants has gradually accumulated in our forests, until a profitable business could again be done in them, had we the Indians among us as laborers. Our negroes cannot be depended upon for discretion and industry, while white laborers regard it as entirely too small business to engage their attention. It is scarcely probable that a business will ever again be done here in them, until the prices shall so far advance as to cause the avarice of our population to overcome their pride.

The fertility of our mountain lands, which chiefly distinguishes this section of Georgia, peculiarly fits it for the spontaneous growth and cultivation of medicinal plants. Our climate intermediate between that of Pennsylvania and Florida, gives us many of the native plants of each, and enables us to cultivate successfully a larger variety.

*Capsicum annuum* grows well here, but not to the perfection of the middle and lower portions of our State.

*Cassia Marylandica* is found in considerable abundance, employed to some extent in domestic practice; not used by our physicians.

*Chenopodium anthelminticum* grows very abundantly in fence corners; old fields are often nearly covered with it; seldom found in the forest; employed in infusion as an anthelmintic under the name of Jerusalem tea. But for the expense of apparatus a good business might be done in the distillation of the oil.

*Chimaphila umbelata* abounds in our forests, but not to the extent it does in portions of middle Georgia. It possesses a sandier soil than ours; much use is made of it "to cleanse the blood." It is sometimes called prince's pine.

*Cimicifuga racemosa* is very abundant along the Chattahoochie River, as also throughout the State; used by the profession in private practice, and freely by the "steamers;" known among the farmers as "rattle weed."

*Frasera Walteri* is very abundant, and frequently offered to druggists under the name of Columbia root; used as a substitute for colomba by the profession as well as in domestic practice.

*Cornus Florida* is a very common tree in our forests, of usually a small size, and is very attractive to the eye when in bloom. Dogwood bark is universally used as a tonic after fevers and intermittents.

*Anthemis cotula* is one of our greatest pests. May-weed, stink-weed, dog-fennel, and wild chamomile are its vulgar names. It completely covers waste lands and the commons around our cities and towns. The bruised herb is said to blister as promptly as cantharides. It is little if at all used here. The infusion gives rise to abortion in females. During the hot summer months it exhales a very offensive odor.

*Eupatorium perfoliatum* is very abundant, and much used in domestic and steam practice.

*Ficus.* The fig grows well with us in some of its more hardy varieties. Our cold winters occasionally cut them down. We grow a fig which, although cut by the frost nearly every winter, is still of so vigorous a habit as to spring up again and bear two crops of very excellent fruit during the season. There are many varieties cultivated in our State. During the past spring, the writer was presented with a box of most delicious figs grown and cured in the lower section of the State. More attention should be devoted to them as an article of commerce. No attempt is made to preserve them here, save in syrup and as pickles for the table, in which forms they are much esteemed.

*Gentiana* is an article brought to us from the country, which is not distinguishable in the root, from the officinal. The plant I have not had an opportunity of examining; we use it in the preparation of the officinal compound. It could probably be obtained in quantities.

*Punica granatum* is much cultivated in the gardens for ornament and use. While in bloom, the beautiful bell-shaped flowers are quite attractive. The fruit is generally esteemed, and its rind, as well as the bark of the root used medicinally.

*Hedeoma pulegioides* covers our hill sides in the open woods and old fields. It may be mown with the scythe and raked like hay. With apparatus for distillation, the oil could be obtnined on a large scale.

*Humulus lupulus* grows finely with us. My garden supplies my retail trade with a quality for which I realize double the prices usually obtained for the commercial article. Little or no attention is given to their cultivation by our farmers.

*Linum.* No attention is paid to the growth of flax except upon a very small scale for the seed used medicinally. Oil is not made at all.

*Lobelia inflata*, although not indigenous to our soil, has been introduced in places among us, and we are occasionally offered both herb and seed.

*Maranta* has never been tested in this section; a few plants obtained by the writer some years ago died, and the experiment has not since been repeated.

*Mel.* Much attention is given to the production of honey for the home market, and small quantities of wax are sent abroad. Wild bees are frequently found in our forests, where they deposit honey in the hollow trees. They are marked, and in due season the tree is felled, and the mangled comb contracted and brought to market. A more than ordinary courage is required for success in this undertaking. The honey is inferior in color and flavor to that of the domesticated bee.

*Mentha piperita* and *viridis* are easily established in our soil, and take the ground completely, producing abundant crops of herb. This plant, in our hot climate, abounds, I think, more largely in the essential oil than in the State of Michigan, where I have observed it in cultivation. There seems every reason to believe that the manufacture of the oil of peppermint would be quite profitable here under judicious management.

*Monarda* is abundant in old fields, and along the road sides. No use is made of it.

*Amygdaline communis.* The almond, both sweet and bitter, have been grown successfully in the middle portion of Georgia. I know of no attempt having been made here.

*Olea Europæa.* Efforts have been made with success to introduce the olive upon our seaboard. It has not been attempted here.

*Ricinus communis* is found occasionally along our road sides. No use is made of it. It is said to drive moles from the gardens where it is grown.

*Terebinthina.* During a few years, the production of turpentine, rosin, and spirits has made some progress in our State. In my own immediate neighborhood the manufacture is as yet quite limited; not more being produced than is consumed in the counties immediately around us. The rosin accumulates on hand, and no arrangements are yet made for shipping it. Our distance from the seaboard precludes the probability of shipping it to advantage. Some experiments have been made in the distillation of the rosin-oil, and it is probable it may be advantageously disposed of in this way. The spirits is distilled from large cast iron pots upon which are luted tin or sheet iron caps, and the vapor is condensed in the ordinary copper worm. No water is introduced with the turpentine. The heat is badly applied, so that a portion of the rosin is often decomposed, and the spirits somewhat contaminated with rosin-oil. When brought to us fresh from the still, the oil of turpentine is almost wholly free from the odor and taste termed terebinthinate, which it acquires by exposure. The flavor of pine bark freshly stripped from the tree, is carcely more acceptible than the recently distilled oil.

*Podophyllum petaltum* is very abundant in low, mosst woods. In many spots the roots may be obtained almost as rapidly as potatoes from the cultivated field, so thickly do they grow. It is much used in domestic and steam practice.

*Prunus Virginiana* is very abundant, and much used, both the bark and berries.

*Sanguinaria* is found abundantly scattered all through our forests. Much used under the name puccoon root.

*Menispermum Canadensis* is very abundant in low grounds along our rivers and small streams; much used as a tonic and alterative. It almost entirely replaces the Smilax officinalis with us.

*Sassafras* overruns our waste lands, and is usually considered a never failing indication of the poverty of the soil upon which it grows. Sassafras tea is a panacea with many; the pith is also much used.

*Senega* is found easily for domestic use. Whether it could be obtained in quantities sufficiently large to make it an article of regular export, I am unable to say.

*Serpentaria* is much more abundant, and could, I think, be made profitable. This is also much used. Two varieties are found and used indiscriminately. The distinctions between the two I have not examined with any care; they are probably contained in the books.

*Spigelia* is very abundant. It is occasionally offered in small lots for sale. It has gone much out of use with us.

*Stillingia* is indigenous, and used to some extent. I have not found it very abundant.

*Stramonium* is our "jimson" weed—a great pest.

*Taraxicum* is never found indigenous in our soil.

*Ulmus.* Our elm does not yield so mucillaginous a bark as that obtained from the Northern States.

*Zingiber* does not bear our climate well, unless it be protected during the winter. Besides the plants named, we have many which are used only in domestic and steam practice. With a few exceptions, we are unable to obtain a home supply of medicinal plants.—*Proceedings of the American Pharmaceutical Association.*

